# ACTH-Modulated Membrane Guanylate Cyclase Signaling System: Origin and Creation

**DOI:** 10.3389/fnmol.2022.929396

**Published:** 2022-08-09

**Authors:** Rameshwar K. Sharma

**Affiliations:** Unit of Regulatory and Molecular Biology, Salus University, Elkins Park, PA, United States

**Keywords:** membrane guanylate cyclase, cyclic GMP signaling pathways, surface receptor, ACTH, transduction modes, signal transduction

## Abstract

The membrane guanylate cyclase (MGC) cellular signaling pathway consists of seven signaling pathways and is critical for the survival of prokaryotes eukaryotes, and highly complex vertebrate organisms. A sequel to the author's earlier comprehensive reviews, covering the field of MGC from its origin to its establishment to the year 2014, this article exclusively deals with the history of its development from the year 1963 to 1987. It narrates the efforts involved in building on small projects, brick by brick, and its emergence from the chasm of disbelief, through steady, continuous work. To make the presentation simple and chronologically continuous, the subject matters of the earlier reviews and publication of these authors have been freely borrowed with appropriate citations.

## Introduction

### Pursuit

Ignited by the fervor of the author's early findings that a common evolutionary link exists between the generation of plant steroids, cardenolides, and the mammalian steroid hormones (Wickramsinghe et al., 1969; Sharma, 1970a,b,c; Kitabchi et al., 1971a,b), he decided to decode this link, and thus, entered the field of hormonal signal transduction. Because cholesterol in both generations was the central precursor molecule, it became the subject matter of the author's research for the next 18 years, from 1969 to 1987.

**Figure 1:**
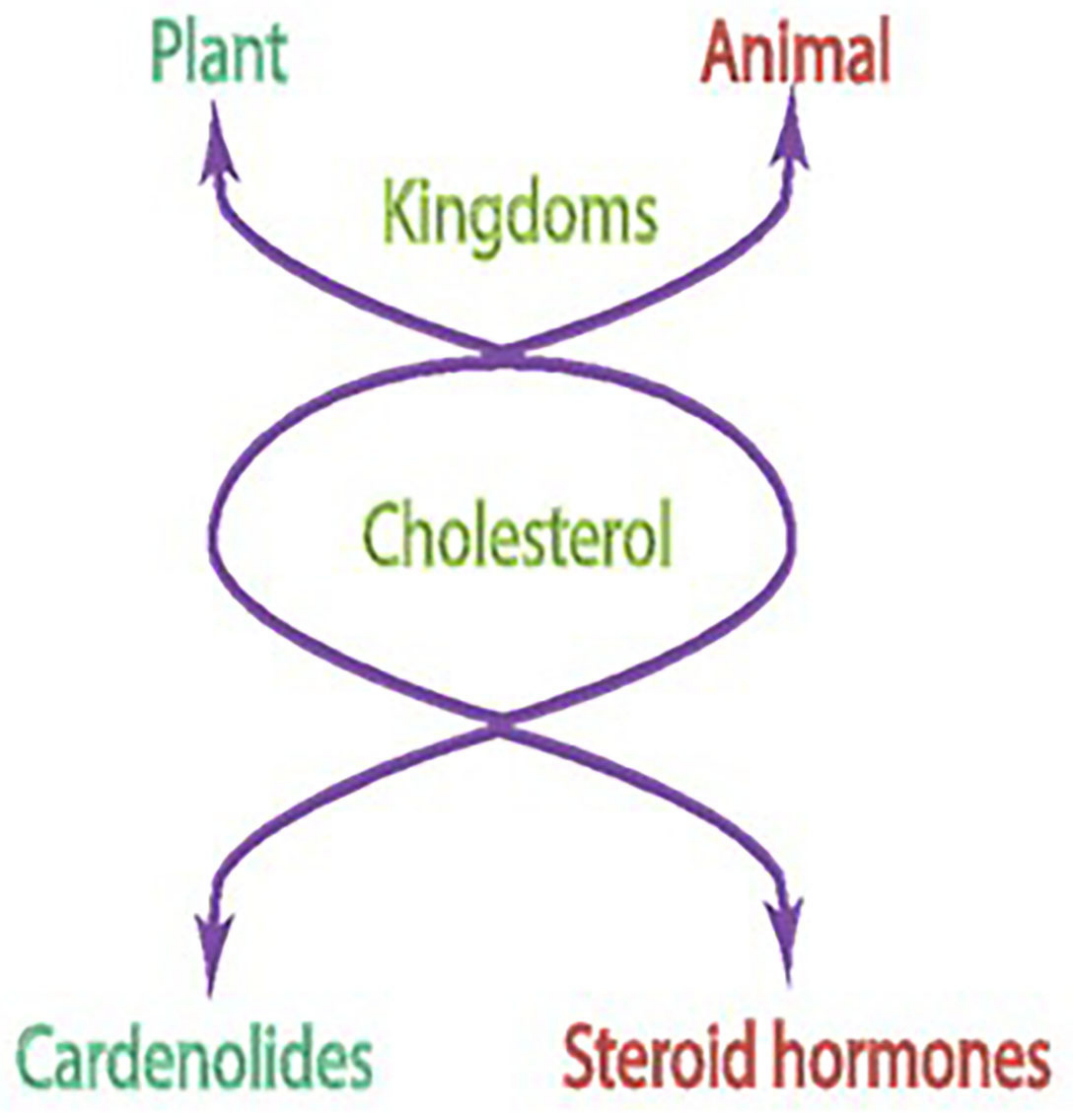
Cholesterol is a common precursor molecule for steroids in animal and plant kingdoms.

The basis of the concept was that in both plant and mammalian kingdoms, biosynthetic pathways of the cholesterol formation are identical; they start from the primordial mevalonic acid molecule (Wickramsinghe et al., 1969; Sharma, 1970b; Kitabchi et al., 1971b). In the evolutionary ladder, a change occurs, however. The mammals develop an extra adrenocorticotropic hormone (ACTH)-dependent branch locked with the primordial cholesterol formation. The locked branch now is a sensor of the ACTH signal, generated in the pituitary gland. Then, the signal is transmitted selectively to the isolated fasciculate cell of the adrenal gland; the cell using its stored cholesterol synthesized from mevalonic acid transforms it into the steroid hormone and cortisol/corticosterone (Kitabchi et al., 1971b). In the plant, in contrast, cholesterol is transformed into cardenolides (Wickramsinghe et al., 1969; Sharma, 1970a,b,c). Outstandingly, in both cases, the targeted site of cholesterol is its side chain: cleavage in mammals and rearrangement in plants. Notably, the initial biosynthetic steps from mevalonic acid to cholesterol stay almost identical in both the cardenolides and in the rat adrenal mitochondria (Wickramsinghe et al., 1969). Only the last biosynthetic steps differ, and solely operate in the fasciculate cell of the adrenal cortex, that is transformation of the (the 20S)-20-hydroxycholesterol to pregnenolone and then to progesterone and to cortisol/corticosterone (Bhacca and Sharma, 1968; Sharma and Hebborn, 1968; Sawhney and Sharma, 1976). In common, in both kingdoms, the cleavage step of the cholesterol sidechain occurs between C-20 and C-22 (Figure 1).

A SPECIAL NOTE is deserved on the development and use of an extremely precise technology to arrive at the stated conclusions. The author picked it up during his tenure at the Worcester Foundation of Experimental Biology (Shrewsbury, Massachusetts, 1968–1969) when collaborating with Dr. E. Caspi's group (Wickramsinghe et al., 1969). Briefly, it involved the application of double isotope labels, 4R-4-^3^H-2-C^14^-mevalonic acid, or/and 7-^3^H-(1,7,15,22,26)-C^14^-cholesterol. In plants, they were fed through painting their leaves and in the fasciculate cells through their incubation in the reconstituted systems. The generated non-radioactive end product, steroid or cardenolide, was then mixed with the experimental product, crystallized, and the ^3^H/C^14^ ratio was evaluated to trace the biosynthetic pathways.

With Caspi's group, the author used this technology for the *Digitalis Lanata* plant cardeneloids, digitoxigenin, tigogenin, and pseudotigogenin; and independently, with his group for the *Cammelia sinensis* plant cardenolides, α-spinasterol and aβ-amyrin (Wickramsinghe et al., 1969).

To attain the original aim, i.e., to decode the LINK between the two kingdoms, the pursuit began. The subject of study was the cholesterol molecule. The experimental models were the isolated fasciculate cell of the adrenal cortex and of the adrenocortical carcinoma 494 cells.

TASK 1. Decode the ACTH-modulated mechanism of the process of steroidogenesis. TASK 2. Apply this knowledge to understand deformities in the cancer cell.

## Task 1

It necessitated the development of a bioassay that detects the physiological concentration of ACTH in the human plasma (Kitabchi et al., 1971a). For this, the isolated system of the fasciculate cells of rats was constructed (Kitabchi et al., 1971a). The adrenal gland was treated with trypsin to selectively remove its zona glomerulosa and the zona reticularis, yielding the sole region of fasciculate. This, in turn, acted as a source of the pure isolated cells, used in the experiments. The cells were physiologically pure, solely synthesizing corticosterone, the counterpart of the human cortisol, detecting as little as 0.5 μunit of ACTH. Conspicuously, there was a small lag period in kicking on the response, demonstrating the auxiliary role of a labile factor. The factor was a protein because its inhibitor blocked the lag. Demonstrating specificity, these fasciculate cells did not respond to other polypeptide hormones, insulin, growth hormone, proinsulin, glucagon, and epinephrine.

In further affirmation, (1) there was a linear relationship between the number of cells, fifty to four hundred thousand, and steroidogenic response to ACTH; (2) in time-dependency, 0 to 180 min interval, the maximal physiological concentration of ACTH (50 μunits) generated corticosterone production in a linear fashion between 6 and 180 min; (3) a definite short lag period (6 min) occurred before the commencement of the linearity in steroidogenesis, a repeatable phenomenon with low (5 μunit) concentration of ACTH.

The physiological purity of the cells was matched with their morphological integrity. The light and electron microscopic analyses demonstrated that the cells embodied the typical fasciculate cell attributes, vascular mitochondria, the excessive cellular content of lipid globules, endoplasmocrine secretion of lipid globules together with the smooth-surfaced endoplasmic reticulum (Kitabchi and Sharma, 1971; Kitabchi et al., 1971a; Sharma et al., 1972).

The applicability of the isolated fasciculate cell assay system in a human study established that the plasma ACTH level in the fasting normal subjects is 2 μunits ± 0.1; the value increases about 6-fold when the generation of cortisol is blocked through the administration of metyrapone (Kitabchi et al., 1971a).

Thus, we had an extra sensitive tool in our possession. This could detect the physiological concentrations of ACTH and could be used in *in vitro* reconstitution studies for direct elucidation of the mechanism through which ACTH controls the steroid hormone cortisol. In turn, we were well-positioned to systematically define the ACTH-dependent physiological processes of stress, fighting infection, and maintaining blood pressure. In addition, we could apply this knowledge to understanding abnormalities in the 494 adrenocortical carcinoma cells.

Cognizant with the developing, at that time, a concept that all hormonal actions occur through the cyclic AMP second messenger system (reviewed in Sharma, 2002), this possibility was also considered in our studies. If applicable, the transformation of the ACTH signal into the production of the steroid hormones would be a three-component-system, consisting of the receptor, the G protein, and the adenylate cyclase. Structurally, the adenylate cyclase is composed of two cytoplasmic domains and two membrane-spanning domains, each of which contains 6 transmembrane spans. As will become clear, this was not the case. The ACTH hormonal signal transduction system was unique physiologically, biochemically, and molecularly.

This conclusion was arrived at through experiments that were conducted under conditions that were as close as possible to the physiological conditions. The five hundred thousand fasciculate cells representing one rat adrenal gland or its counterpart, the adrenocortical carcinoma 494, were used in the reconstitution studies.

The findings of these studies were as follows (reviewed in Sharma, 2002):

- ACTH stimulates the biosynthetic step of steroidogenesis from (the 20S)-20-hydroxycholesterol; this step is dependent on protein synthesis and is rate-limiting. Yet, the cyclic AMP does not mimic this stimulatory action of ACTH (Sharma, 1973, 1974; Sharma and Brush, 1974). Thus, cyclic AMP is not the physiological second messenger of ACTH challenging the prevailing universality of the “cyclic AMP second messenger concept”.
- Accordingly, ACTH in the carcinoma cells inhibits steroidogenesis, yet cyclic AMP does not.
- In a bell-shaped curve, the physiological concentration of ACTH, 2.5–10 μunits, does not raise the level of cyclic AMP but does stimulate steroidogenesis. Yet, it stimulates the peak synthesis of cyclic GMP with a concomitant rise in corticosterone. Thus, cyclic GMP, not the cyclic AMP, is the physiological mediator of the ACTH hormone.
- A surprising finding was that supra-physiological concentrations, 10–100 μunits, of ACTH, indeed, stimulate cyclic AMP levels in a concentration-dependent manner with a concomitant rise of the corticosterone level. Notably, under these conditions, there was no rise in the cyclic GMP levels.

Taken together, these studies established that the physiological second messenger of ACTH is cyclic GMP thus, negating the concept that cyclic AMP is the universal second messenger of hormonal action and demonstrating that cyclic AMP is only a pharmacological transmitter of the ACTH response.

The above conclusions were in accord with the independent findings of other groups. They used different techniques and also reported that their data does not support the second messenger role of the cyclic AMP for the ACTH hormone (Davis and Garren, 1968; Montague and Howell, 1973; Richardson and Schulster, 1973; Sharma et al., 1976). Briefly, Moyle et al. (1973) in isolated adrenal cells found a lack of correlation between the ACTH–dependent steroidogenesis and cyclic AMP production. Using isolated adrenal cell column perfusion technology, Hudson and McMartin found that submaximal steroidogenic concentrations of ACTH do not release cyclic AMP into the perfuse (Hudson and McMartin, 1975). After ACTH stimulation of sheep adrenals *in vivo*, Espiner et al. (1974) did not detect cyclic AMP output at submaximal steroidogenic concentrations of ACTH. Honn and Chavin (1975) demonstrated that ACTH elevates cyclic GMP levels without a rise in cyclic AMP in the reptile adrenals.

Armed with the tools of the model isolated cell lines of the fasciculate and the adrenocortical carcinoma 494 cells, with the techniques of double-labeled ^3^H/C^14^ radioisotopes, and with the information that ACTH control of steroidogenesis is indirect, occurring through the mediation of its second messengers, cyclic GMP, and cyclic AMP, we were in a good position to define the ACTH-dependent rate-limiting step of steroidogenesis (Wickramsinghe et al., 1969; Sharma, 1970a,b,c). Because none of the biosynthetic steps between cholesterol and (the 20S)-20-hydroxycholesterol were affected by ACTH, the transformation of (20S)-20-hydroxycholesterol to the final product corticosterone was investigated.

The results (Bhacca and Sharma, 1968; Sharma and Hebborn, 1968; Sawhney and Sharma, 1976) established that cleavage of the (the 20S)-20-hydroxycholesterol to corticosterone is the rate-limiting step and it is controlled by ACTH. The reaction occurs at the plasma membrane of the normal cells but does not occur in the malignant cells (Sawhney and Sharma, 1976). The cyclic GMP mimics the ACTH-dependent physiological effect and the cyclic AMP, the pharmacological effect. Notably, both cyclic nucleotides are unable to mimic the ACTH response in the carcinoma cells. These results were consistent with the conclusions that cyclic GMP and cyclic AMP are their respective physiological and pharmacological second messengers; they are generated inside the cell in response to the ACTH signal at the plasma membrane; the lesion/s in the carcinoma cell membrane terminates the ACTH, yet retains the second messenger activities.

To explain the bell-shaped configuration of the cyclic GMP curve in response to the ACTH concentration between one and 10 μunits, the following mathematical rate equation was derived from the ACTH-dependent membrane guanylate cyclase (Sharma, 1976). It has two sites with different affinities, *K*_1_, and *K*_2_; the first molecule of ACTH activates the enzyme activity while the second molecule blocks it.


(1) $$\begin{array}{r} V=\frac{V_{max}SA}{K_{1}Km+\left(K_{m}+S\right)A+K_{m}A^{2}/K_{2}} \end{array}$$

*S* and *A* represent the concentrations of GTP and ACTH, respectively, and *K*_1_ and *K*_2_ are equilibrium constants. Hence, the enzyme combines with the second molecule of ACTH with greater affinity than with the first one; the binding of the first ACTH molecule allosterically modifies the structure of the membrane guanylate cyclase.

The fact that the physiological concentrations of ACTH elevated cyclic GMP levels without affecting the level of cyclic AMP indicated that cyclic GMP plays a critical role in mediating ACTH-stimulated steroidogenesis in the isolated fasciculate cell. Also, cyclic GMP may also be responsible for the formation of/or processing of specific mRNA that in turn may be translated into a process that is also cyclic GMP-dependent. However, at higher concentrations (more than 10 μunits) ACTH stimulates only the adenylate cyclase system. The latter stimulated system would be responsible for the synthesis of cyclic AMP, that in turn, through cyclic AMP-dependent protein kinase, may cause translation of the appropriate mRNA. Notably, at the time, the year 1976, cyclic GMP-dependent protein kinase was not known in the isolated fasciculate cell; however, at a later date, it was purified and characterized from the adrenal cortex (Ahrens and Sharma, 1979; Ahrens et al., 1982). Accordingly, the physiological concentration of ACTH generated cyclic GMP, activated cyclic GMP-dependent protein kinase, and finally produced the steroid hormone, corticosterone. Then, it was possible to prove that cyclic GMP-mediated steroidogenesis is mediated by cyclic GMP-dependent protein kinase.

In this manner, cyclic GMP originally detected in the rat urine by Ashman et al. (1963) was now qualified to be the second messenger of the polypeptide hormone ACTH. According to this concept, in sequential steps ACTH secreted from the pituitary through circulation was transported to the target fasciculate cell of the adrenal cortex; therein, it targeted the plasma membrane, generating intracellularly second messenger cyclic GMP; that in turn, through a chain of reactions produced corticosterone. The findings also demonstrated how this signaling was abnormal in the adrenocortical carcinoma 494 cells.

As stated, the second messenger role of cyclic GMP in the ACTH-modulated steroidogenesis was determined indirectly through the reconstituted *in vivo* cell studies, isolated fasciculate and carcinoma 494. To assess it in a direct and unequal fashion, the studies beyond the year 1976 were directed toward dissecting the components of these cells. Included in these studies was the component of the cyclic GMP-dependent protein kinase.

In the above-described studies, the level of cyclic GMP was measured through radioimmunoassay. To affirm these results, our group developed an alternate direct, yet equally sensitive assay (Perchellet et al., 1978). Based on the highly specific binding property of *E. coli* polypeptide chain elongation factor Tu (EF-Tu) with guanosine diphosphate (GDP), it included the conversion of cyclic GMP to 5′-GMP by phosphodiesterase (E.C.3.1.4.1) and the transfer of ^32^P from [γ-^32^P]adenosine triphosphate to GMP to yield [β-^32^P]GDP by GMP kinase. There was no cross-reactivity with other cyclic nucleotides, including, cyclic AMP and cyclic IMP. The experiments where the cyclic GMP was measured through its radio-immunoassay were done side-by-side. The results were remarkably identical with both assay techniques.

They proved that, indeed, cyclic GMP was the physiological and cyclic AMP the pharmacological mediator of ACTH in the process of steroidogenesis. In the former case, there was the connected rise of cyclic GMP protein kinase, yet no rise of the cyclic AMP-dependent protein kinase. In the latter, the reverse was the case (Sharma and Sawhney, 1978).

The investigation moved on to define the physiological composition of the membrane guanylate cyclase transduction system (Perchellet and Sharma, 1979). Mimicking the natural cellular environment, the reconstitution study showed that the concentration of 2.5 mM Ca^2+^ was obligatory for the physiological levels of ACTH to generate the production of cyclic GMP and cause steroidogenesis in the isolated fasciculate cell. The Ca^2+^ alone did neither stimulate cyclic GMP production nor the process of steroidogenesis. Thus, Ca^2+^ and cyclic GMP are co-messengers in the ACTH-dependent steroidogenic process.

This fact taken together with the earlier one that cyclic GMP-dependent protein kinase activity concomitantly rises following the steroidogenic step, a model depicting the role of Ca^2+^ and cyclic GMP in ACTH-activated steroidogenesis was proposed (Figure 4 in Perchellet and Sharma, 1979).

## Model

**Figure 2:**
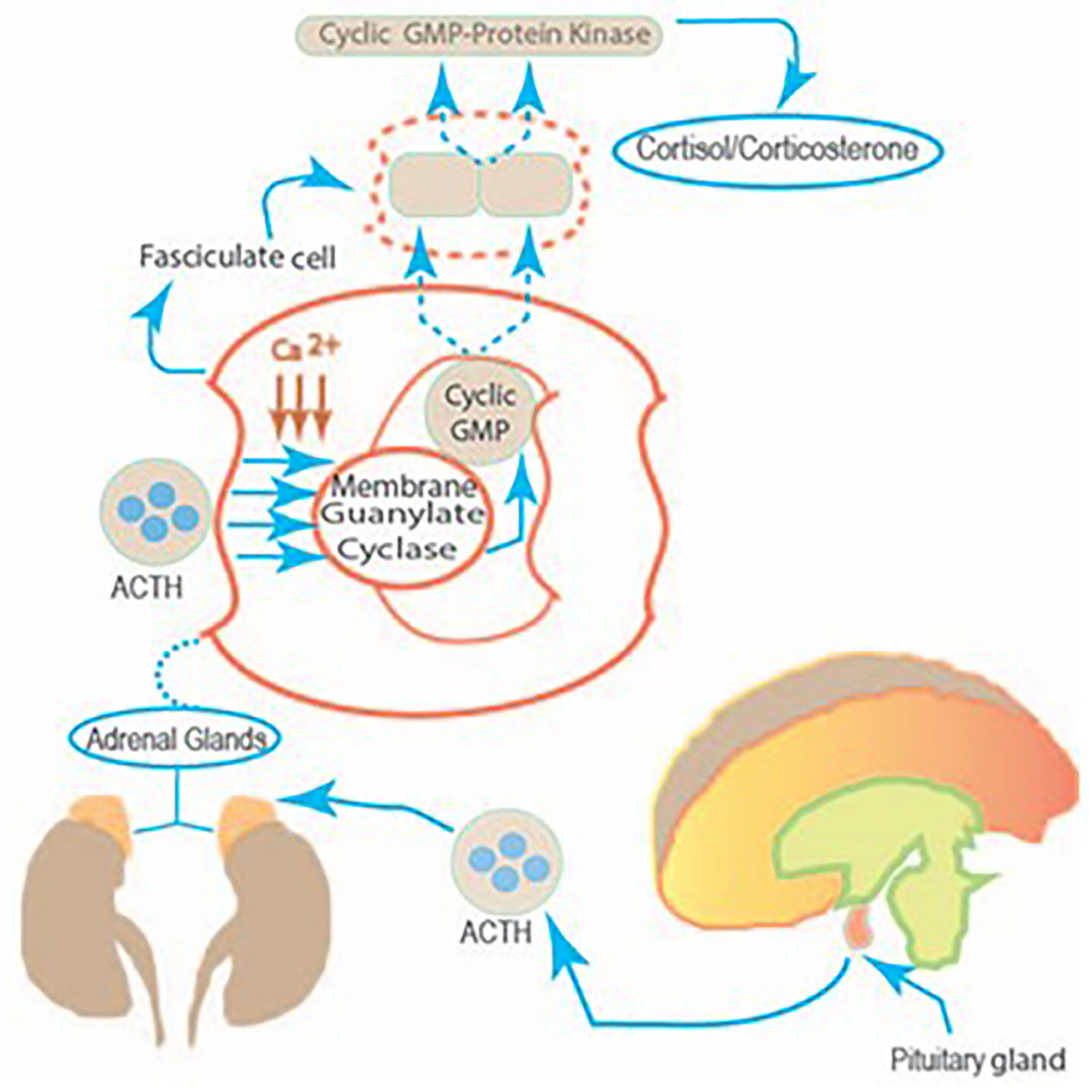
The linkage between the pituitary gland and the fasciculate cell of the adrenal cortex. The molecular mechanism by which two ACTH co-messengers, Ca^2+^ and Cyclic GMP modulate steroidogenesis. The stimulus from the corticotropin-releasing factor, generated in the hypothalamus, produces corticotropin-releasing factor (CRF) in the anterior lobe of the pituitary. Therein, CRF controls ACTH release. This, in turn, targets the fasciculate cell of the adrenal cortex, causing a chain of molecular reactions and generating cortisol production.

ACTH binds to its receptor located on the plasma membrane of the fasciculate adrenocortical cell. The Ca^2+^ regulates the transduction of information between hormone receptors and membrane guanylate cyclase. This leads to the increase in cyclic GMP which in turn, activates the cyclic GMP-dependent protein kinase activity, leading to the translation of a hypothetical preexistent mRNA. The synthesized protein controls the entry of cytoplasmic cholesterol into the mitochondrial precursor pool of cholesterol. The ACTH guided by these steps transforms this pool of cholesterol into the steroid hormone cortisol/corticosterone (Figure 2).

The validity of this model was supported by the purification of the adrenocortical cyclic GMP-dependent protein kinase from the bovine adrenal cortex (Sharma and Ahmed, 1976; Ahrens and Sharma, 1978; Ahrens et al., 1982). Notably, at that time, not only the identity of a cyclic GMP-dependent protein kinase was missing, but its existence was denied in the mammalian systems (Gill and McCune, 1979).

It was established that the Mr of the cyclic GMP-dependent protein kinase enzyme is 145,000; it is composed of two identical subunits each with Mr of 75,000. The enzyme self-phosphorylates; the stoichiometry of cyclic GMP binding is two molecules per holoenzyme, one to each of its subunits. The Ca^2+^-modulated proteins, calmodulin or troponin C, markedly stimulate the maximal velocity of the enzyme. The steroidogenic potential of cyclic GMP and its analogs correlates closely with their ability to stimulate cyclic GMP-dependent protein kinase.

With the recognition of this puzzle piece, the ACTH signaling model was refined. Here, the two generated co-messengers of ACTH, Ca^2+^ and cyclic GMP activate the cyclic GMP-dependent protein kinase, this, in turn, accelerates the process. The mechanism of cyclic GMP activation of the protein kinase is that two molecules of cyclic GMP bind to the protein kinase holoenzyme, one to each of its subunits. Thus, the cyclic GMP-dependent protein kinase was no more a missing piece of the ACTH-modulated steroidogenic process, it was a part of it. It sub-served the activities of two complementary physiological second messengers of ACTH, cyclic GMP, and Ca^2+^.

The discovery and molecular characterization of the cyclic GMP-dependent protein kinase powerfully impacted not only the field of hormonally-modulated membrane guanylate cyclase locked with the process of steroidogenesis but also corrected its molecular identity proposed by other laboratories as narrated below.

Originally, in 1971, Greengard's group discovered that the fat bodies of silk moth pupae and larvae contain exclusively cyclic GMP-dependent protein kinase activity (Kuo et al., 1971). Subsequently, the group identified this protein kinase in the mammalian cerebellum (Schlichter et al., 1980), expanding its presence from the MOTH kingdom to the mammalian. Its molecular identity from bovine lung and heart demonstrated that its Mr ranged from 145,000 to 165,000 (De Jonge and Rosen, 1977; Gill et al., 1977; Lincoln et al., 1977). The protein was composed of two equivalent subunits (Takai et al., 1975). Like the previously characterized cyclic AMP-dependent protein kinase holoenzyme, it was also a holoenzyme; each of its subunits was of a regulatory and a catalytic unit. Upon binding cyclic GMP, the holoenzyme underwent dissociation into regulatory and catalytic subunits (Miyamoto et al., 1973; Van Leemput-Coutrez et al., 1973; Kuo et al., 1974; Kuo, 1975; Kobayashi and Fang, 1976).

Ours and other groups, independently, showed that the assignment of the latter characteristic to the enzyme was flawed (Takai et al., 1976). The cyclic GMP-dependent protein kinase does not undergo dissociation upon binding into the stated subunits (Takai et al., 1976; Lincoln et al., 1977). It binds two molecules of cyclic GMP, one with each of its subunits (Sharma and Ahmed, 1976; Ahrens and Sharma, 1978; Kuroda and Sharma, 1982). Thus, the paradigms of signaling between the two systems, cyclic AMP and cyclic GMP are totally different.

Being confident now on the second messenger role of cyclic GMP in the ACTH-modulated process of steroidogenesis, our research advanced from the reconstituted isolated fasciculate cell to a cell-free system. Before the author recounts it, he describes its status in the cell signaling field at that time.

## The Existence of the Hormonally-Dependent Membrane Guanylate Cyclase in Any Mammalian System Was Denied

The denial was aggressively driven by an elite group-linked network of investigators (Goldberg and Haddox, 1977; Murad et al., 1979). Their collective, yet diffused, reasoning was that the hormone-dependent membrane guanylate cyclase enzyme does not exist in nature; the rise of cyclic GMP observed in certain biological systems is artifactually caused by non-hormonal signals, like hydroxyl radical, hydrogen peroxide, lipids, and unsaturated fatty acids, oxidants, nitric oxide, and a variety of other nitric oxide generating compounds, such as nitrosoamines, including cigarette smoke (Wallach and Pastan, 1976; Arnold et al., 1977a,b; Katsuki et al., 1977a,b; Mittal and Murad, 1977a,b; Mittal et al., 1977; Murad et al., 1978a,b; Waldman et al., 1982). It was proposed that the underlying mechanism for the nitrite–generating compounds was the same, *via* nitric oxide gas (NO). Supporting this proposal, remarkably, the Murad's group published purification of a membrane guanylate cyclase from rat liver; yet it was not hormonally responsive. In contrast, NO targeted its catalytic site, its Mr was 80,000, and its substrate was Mn-GTP (Waldman et al., 1982); and, this membrane guanylate cyclase was present in all tissues ranging from peripheral to the central nervous system (Katsuki et al., 1977a,b). In 1981, Murad's group reported purification of the same enzyme from rat lungs but they revised its molecular weight to 150,000, a dimer consisting of two identical, Mr 72,000, subunits. The purified enzyme could be activated by nitric oxide, indicating that this compound interacts directly with the enzyme. Their later studies asserted that this guanylate cyclase could also be activated by other agents, hydroxyl radicals, and unsaturated fatty acids. In a subsequent 1986 publication, reporting similar results, yet, purified through immunoaffinity chromatography, the Murad's group again concluded that the rat lung enzyme inherits the pure soluble guanylate cyclase characteristics; its molecular weight is 150,000, it consists of two subunits with molecular weights of 82,000 and 70,000. Consequently, this group published similar characteristics of the purified rat liver, its molecular weight was 150,000; unreported was its subunit composition; yet the liver enzyme could be activated by nitroprusside, nitric oxide, arachidonate, linoleate, oleate, and superoxide dismutase (Braughler et al., 1979).

The conclusion of these erroneous claims was that the membrane guanylate cyclase was a non-specific enzyme that underwent regulation *via* the oxidation-reduction potential of the biochemical reactions (Murad et al., 1979). Also, at the time, the only known cyclic GMP-dependent cellular component was cyclic GMP-dependent protein kinase, yet not even one of its specific, distinct from that of the cyclic AMP-dependent protein kinase substrate was known. Hence, the revised consensus was that the cyclic GMP-dependent system lacked specificity and acted as a sub-servant to the cyclic AMP system. Therefore, no distinct hormonally dependent membrane guanylate cyclase or cyclic GMP-related specific signaling system existed in any of the biological systems and cyclic GMP had no second messenger role in cellular signaling (Gill and McCune, 1979).

### Direct Membrane Guanylate Cyclase Studies in the Cell-Free System

To solve the confusion between the existence of the hormone (ACTH)-dependent membrane guanylate cyclase and its non-hormonal and non-specific soluble form, we used our model systems of the isolated fasciculate cell and the isolated adrenocortical carcinoma 494 cells. Their particulate fractions constituted the membranous and the soluble, the soluble form. The experiments were conducted side-by-side; and for the first time, it was possible to precisely compare their relative contents and their signaling properties from the same cells. The conclusions, summed below, settled that in the adrenal fasciculate and the adrenocortical carcinoma cells the membrane guanylate cyclase is solely coupled with the ACTH-dependent steroidogenesis, yet the soluble form is not (reviewed in Sharma, 2002).

1. The comparable cellular contents of the membrane vs. soluble forms of the guanylate cyclase are 80 vs. 20%.
2. Only the membrane form responds to the physiological concentrations of ACTH in the generation of cyclic GMP. The curve is bell-shaped; the 10^−11^ M brings it to the basal level.
3. The sodium nitroprusside or ascorbic acid only stimulates the soluble form.
4. The dithiol reagent inhibits the soluble form, yet has no effect on the membrane form.
5. Tuffstin, a four amino acid peptide, only stimulates the soluble form.
6. The dithiothreitol and N-ethylmaleimide, the sulfhydryl reagents, have very different types of effects on the membrane and soluble forms. The low concentrations of dithiothreitol (up to 2 mM), markedly stimulate the soluble, yet not the membrane form. N-ethylmaleimide inhibits the membrane but stimulates the soluble form.

After settlement that almost sole form in the intact isolated fasciculate cell is the membrane form of the guanylate cyclase and it is distinct from the soluble form, we endeavored to dissect the two co-messenger, Ca^2+^ and cyclic GMP, biochemical components of the ACTH-modulated membrane guanylate cyclase system (Nambi et al., 1982c). For this task, we used the phenothiazone drug and chloropromazine. It selectively targets its Ca^2+^ site and blocks its interaction with the Ca^2+^-receptor protein, calmodulin.

The conclusions were that chloropromazine, indeed, blocks the ACTH-modulated steroidogenesis; yet, it has no effect on the ACTH-binding step. Thus, in accordance with our previous conclusions, the guanylate cyclase functional system is composed of separate receptor and catalytic components; the Ca^2+^ *via* its Ca^2+^ binding protein, calmodulin, brings them together and makes the system biochemically active to catalyze the guanylate cyclase and generate its second messenger cyclic GMP. In this manner, Ca^2+^ and cyclic GMP serve as co-messengers of the ACTH-modulated membrane guanylate cyclase signaling system. Thereby, two independent forms of guanylate cyclase signaling systems were established in the fasciculate cell of the adrenal cortex and in the adrenocortical carcinoma 494 cell systems. One was membrane-bound and hormonally-modulated, the other was soluble and non-specifically activated. The ACTH-modulated signaling pathway originating from the tiniest gland in the brain, the pituitary, to the sole fasciculate cell in the adrenal cortex was established (Figure 1).

## In This Manner, The Dogma That Hormonally-Dependent Membrane Guanylate Cyclase Does Not Exist in Nature Was Shattered

A new cellular signal transduction field evolved. Ironically, in a lively scientific commentary the main group that consistently challenged its existence, later falsely claimed its foundation (Sharma, 1988). Besides being impure, consisting of at least 13 protein contaminants, the claim-based membrane guanylate cyclase was stimulated by hemin, a biochemical characteristic not inherited by the membrane guanylate cyclase, yet solely inherited by the soluble form of the membrane guanylate cyclase (Waldman et al., 1985).

The establishment of the ACTH-modulated membrane guanylate cyclase signaling pathway, originating in the pituitary gland of the brain and terminating at its target site in the isolated fasciculate cell of the adrenal cortex, at the morphological, physiological, and biochemical levels made it possible to compare it with its counterpart adrenocortical carcinoma 494 cells (Sharma, 1976, 1978, 1985; reviewed in Sharma and Criss, 1978). There were deformities at all levels, briefly summarized here; they were in accordance with the earliest concepts proposed by Claude Bernard and Charles Huggins. “... There must be controlling elements within the composition of fluids that bathe the cells of a multicellular organism”. These thoughts were expanded in the book “Endocrine Control in Neoplasia” which the author edited together with Dr. Wayne E. Criss (Sharma and Criss, 1978), describing the lesions inflicted by carcinogenesis in a variety of cells.

Enlarging on these thoughts, the following sections demonstrate how these ACTH studies directly impacted the general cellular signal transduction pathways. They resulted in the discovery of a protein kinase, SPK 380 that controls the protein synthesis; a protein kinase, another AUT-PK 500 that only exists in the cancer cells, and α_2α*D*_-adrenergic receptor that is linked with the epinephrine regulation.

## The Adrenocortical Carcinoma 494

In 1971 our group established the rat colony bearing this cancer from the seed rats generously donated by Ney's group. This colony was maintained for 20 years (to the year 1989) in our laboratory; sadly the colony ceased to exist in the world when we were unable to sustain it for the lack of funds. Originally, this colony was developed by Snell and Stewart (1959) in sequential publications, both Ney's (Ney et al., 1969) and the Snell and Stewart's (Snell and Stewart, 1959) groups originally demonstrated that, in contrast to what occurs in the normal adrenal cortex, neither ACTH nor cyclic AMP stimulates the formation of corticosterone (reviewed in Sharma, 1976, 1978; Sharma and Criss, 1978).

In a systematic dissection, in contrast to the normal isolated fasciculate cell, our group found the following deformities in the adrenocortical carcinoma 494 cell system at the morphological, pharmacological, biochemical, and molecular levels.

### Morphology

The ultrastructural characteristics of these cells were that they were round to oval and uniform in size; solidly packed and connected with inter-digitation of numerous villi projecting the cell membranes; no one cell was covered with a basal lamina; lipid granules were rare but when present, they were not surrounded by the smooth-surface endoplasmic reticulum (Sharma and Hashimoto, 1972).

### Pharmacology

The cells had no physiological and pharmacological ACTH-responsive membrane guanylate cyclase and cyclic AMP second messenger signaling systems; yet, they contained an autonomous and powerful corticosterone generating system. Albeit, at the supra-pharmacological ACTH levels it contained a barely functional membrane guanylate cyclase transduction system (Perchellet and Sharma, 1977; Sharma et al., 1977).

### Biochemistry

Direct studies demonstrated that the cells, not only lacked the second messenger cyclic AMP and cyclic GMP signaling networks, but they also were devoid of their next network components, cyclic GMP-dependent, and cyclic AMP-dependent protein kinases.

Thus, the biochemical conclusions were that the adrenocortical carcinoma 494 cells have generated an autonomic ACTH signaling machinery; it neither contains the normal second messenger signaling components of the membrane guanylate cyclase and cyclic AMP, nor of cyclic AMP-dependent protein kinase and cyclic GMP-dependent protein kinase, yet it generates huge amounts of ACTH with the concomitant production of an overabundance of corticosterone. Thus, this autonomously controlled network functions to manifest the trophic activity of the steroid hormone, corticosterone. This activity is to sustain the survival of the cancer cell. Curiously, the normally sized adrenal gland weighs ~30 mg, in contrast, one tumor nodule weighs 5–10 g; the bellies of the cancer-bearing rats are massively ballooned.

Ignited by these findings was a conscious awakening that the solution to this puzzle may reside in the cyclic nucleotide-independent protein kinase machinery present in the neoplastic cell. This defective tumor-evolved machinery will powerfully enhance the trophic effect of the steroid hormone corticosterone. It will be, however, absent in the normal cell. In addition, it was hypothesized that the normal fasciculate cell may contain its own unique protein kinase that keeps it non-malignant. The studies briefly narrated below, supported these concepts and exhilarated the mind. Two novel protein kinases with the following inquiries were discovered.

The inquiries were (1) Does the adrenal cortex contain a protein kinase that is locked with the initiation of the protein synthesis? (2) Does adrenocortical carcinoma 494 possess a unique protein kinase?

## Task 1

The self phosphorylating protein kinase 380 (SPK 380) was purified to homogeneity; biochemically and functionally characterized from the bovine adrenal cortex (Kuroda and Sharma, 1982, 1983; Kuroda et al., 1982a,b). The choice of the bovine species was based on the availability of a sufficient amount of the source material.

SPK 380 is a holoenzyme, consisting of three identical subunits of M_r_ 120,000. It is the self-phosphorylating protein kinase. It catalyzes the phosphorylation of its own histidine residue on τ-the amino group; this self-phosphorylation feature is reversible and unique to itself. In the presence of ADP and Mg^2+^, the phosphate bound to the histidine residue is transferred to ADP, resulting in the formation of ATP. The SPK 380 specifically catalyzes the phosphorylation of serine residue of the α-subunit of eukaryotic initiation factor-2 (eIF-2α). Thus, inheriting a unique feature, a histidine protein kinase catalyzes the phosphorylation of the serine residue of another protein, eIF-2α. Functionally, in the reticulocyte lysate, SPK 380 by phosphorylating eIF-2α inhibits protein synthesis.

Significantly, focusing on the last property, SPK 380 is different from the two other cyclic nucleotide-independent eIF-2α protein kinases, hemin-controlled repressor (HCR) and double stranded-RNA activated inhibitor (dsRI) that also inhibit the initiation of protein synthesis. In contrast to these, its activity is independent of hemin and dsRI (discussed in Kuroda et al., 1982b).

Additional evidence for the SPK 380 (or its very close structurally-related protein kinase) role in protein synthesis was provided by its presence in the poly(A) mRNAs contained in the polysome fractions of the adrenal cortex (Moore and Sharma, 1980).

Thus, with the discovery of SPK 380, a new protein kinase feature arose. The protein kinase was no longer only a serine protein kinase that solely transmitted the signaling activities of cyclic AMP and cyclic GMP; or of eIF-2α regulating translational activities of HCR and dsRI. It also existed with a novel feature of being itself the Histidine Protein Kinase and eIF-2α-serine kinase that regulated the mammalian cell protein synthesis.

**Figure 3:**
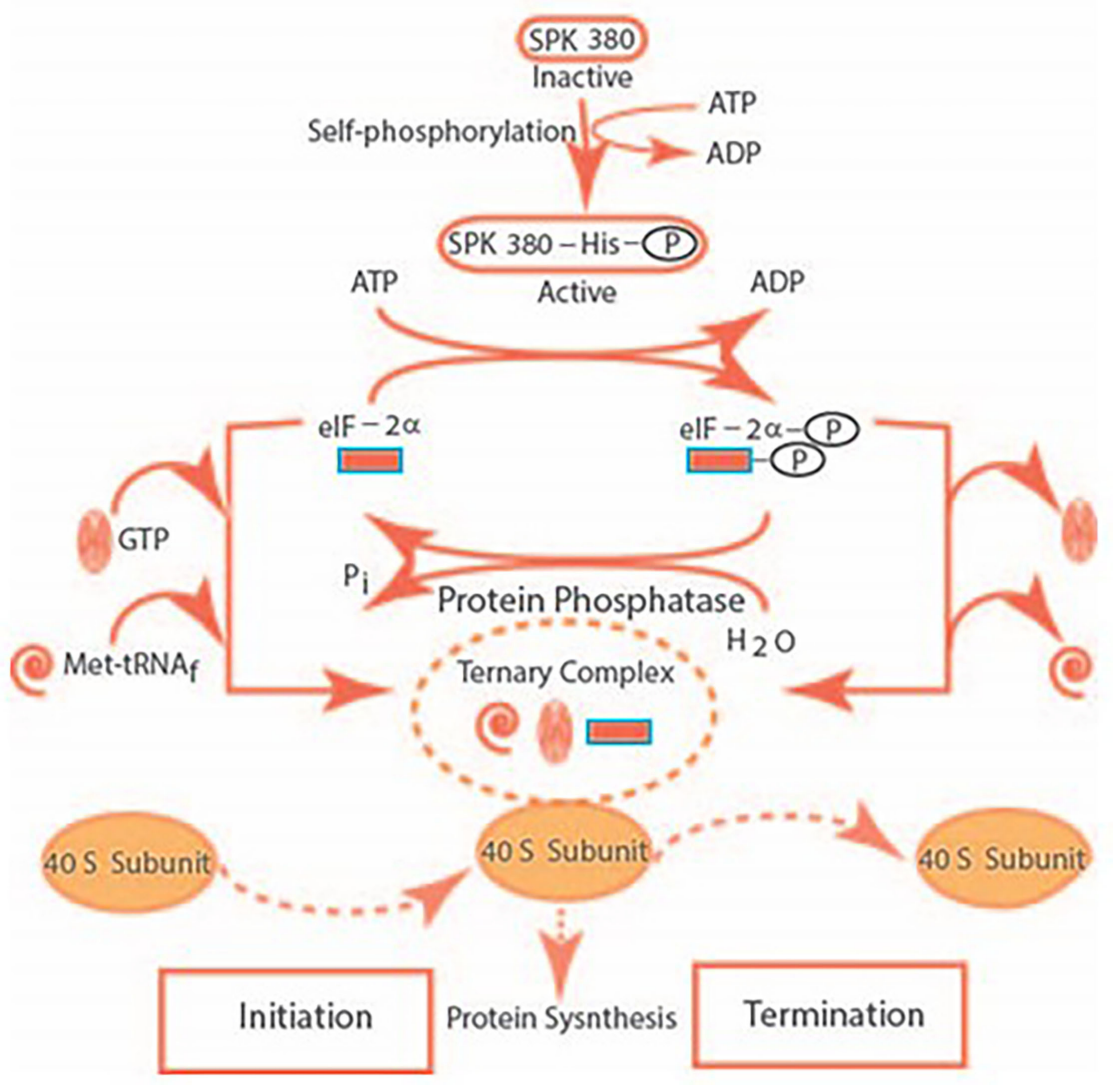
The SPK self-phosphorylates its histidine residue and transforms itself from its inactive to the active form. It catalyzes the phosphorylation of the serine residue of eIF-2α. The eIF-2α-P dissociates the ternary complex of GTP, Met-tRNA_f_, and the 40S ribosomal subunit. It results in the termination the of initiation of the protein synthesis. The ternary complex is restored after de-phosphorylation of eIF-2α, thus, restoring protein synthesis.

A model for the mechanism of translational control by SPK 380 is depicted below in the figure, reformatted and advanced from Sharma (1985) (Figure 3).

The elegance of this model was that the de-phosphorylation and the phosphorylation of eIF-2α represented the TURN “ON” and TURN “OFF” SWITCHES of the cellular protein synthesis. Thus, it solved the age-old puzzle of how polyamines, including tropic hormones—ACTH, Luteinizing, Thyroid Stimulating, Corticotropin-Releasing—are generated and secreted by the anterior pituitary; in turn, target and regulate their respective endocrine organs. Additionally, their eIF-2α-modulated “ON” and “OFF” switches are precisely tuned.

Any defect, affecting this switching mechanism will cause cellular abnormality, including cancer. Here, the endocrine cell embodies the unlimited protein-synthesizing machinery. The case in point is that the adrenocortical carcinoma 494 cells are the counterpart of the fasciculate cell of the adrenal cortex and it inherits the cancer deformities.

The validity of this hypothesis was attested by experimentation (Kuroda et al., 1982b).

The SPK 380 phosphorylated eIF-2α and polyamines—putrescine, spermidine, and spermine—inhibited phosphorylation of eIF-2α. The inhibition was concentration-dependent, with an order of potency: spermine > spermidine > putrescine. Since polyamine is basic, it was a possibility that the effect was artifactual. It, however, was not the case. Other basic peptides—polylysine and polyarginine—did not block SPK 380-dependent phosphorylation of eIF-2α.

Given the pieces of evidence that (i) the rate-limiting enzyme in the synthesis of putrescine, the precursor of the polyamines spermidine and spermine are, ornithine decarboxylase, (ii) its activity increases in parallel with the formation of putrescine and polyamines in rapidly growing tissues, (iii) in similarity with cases of the trophic hormone stimulation of the adrenal gland, thyroid, and gonads, it is possible that the activated SPK 380-dependent phosphorylation of eIF-2α is the determinant factor in the normal protein synthesis. Yet, it is broken down in their cancer counterpart organs.

Focusing on the adrenocortical carcinoma 494 cells, a brief description of the biochemistry of the Two Autonomously Controlled Systems is provided (Sharma et al., 1980; reviewed in Sharma, 1983; Ganguly et al., 1984; Majumdar et al., 1985, 1989).

## One

Autophosphorylating Protein Kinase 500 (AUT-PK 500). Its purity and its self-phosphorylation characteristic were established by the criteria of (a) one- and two-dimensional polyacrylamide gel electrophoresis, (b) immuno-electrophoresis, and crossed- immuno-electrophoresis with an immunoglobin produced against the enzyme, and (c) by the radioautography analysis of the phosphorylated and radio-iodinated enzyme.

Consisting of two identical subunits, 250,000-dalton, the AUT-PK 500 holoenzyme is a unique protein. Its activity or modulation is independent of the cyclic nucleotides, calcium, and also of another tumor-specific cyclic AMP-binding protein kinase, AUT-PK 85 (Shanker et al., 1979). It also is distinct from other cyclic nucleotide-independent protein kinases—the casein kinases I and II, the heme-controlled repressor, the double-stranded RNA-activated inhibitor, protease-activated kinases I and II and, the previously described SPK 380.

The AUT-PK 500 catalyzes the phosphorylation of its serine residue; the terminal phosphate of ATP is specifically used in this reaction. Anti-AUT-PK 500 blocks the self-phosphorylation reaction of the enzyme. Significantly, in contrast to SPK 380, polyarginine has no effect on the autophosphorylation of AUT-PK 500.

Immunofluorescent search using rabbit anti-AUT-PK 500 IgG was made to detect the presence of AUT-PK 500 in the cultured colony of the adrenocortical carcinoma 494 cells. Its distribution was in vesicular cytoplasmic and perinuclear layers. The refined search demonstrated that the AUT-PK 500 resided exclusively in the membrane-bound ribosomal protein of Mr 31000, recognizing it to be its specific substrate; this finding was attested by screening through the use of its monoclonal antibody. Importantly, its level of presence was almost undetectable in the normal tissues—liver, spleen, testis, kidney, ovary, adrenal gland, pancreas, heart, lung, and pituitary. It became 100-fold elevated in the adrenocortical carcinoma 400, 50-fold in the four chemically-induced rapidly growing hepatomas, 30-fold in the chemically-induced mammary carcinoma, 20-fold in the cultured hepatoma cell line, and a 30-fold elevation in the pituitary tumor.

**Figure 4:**
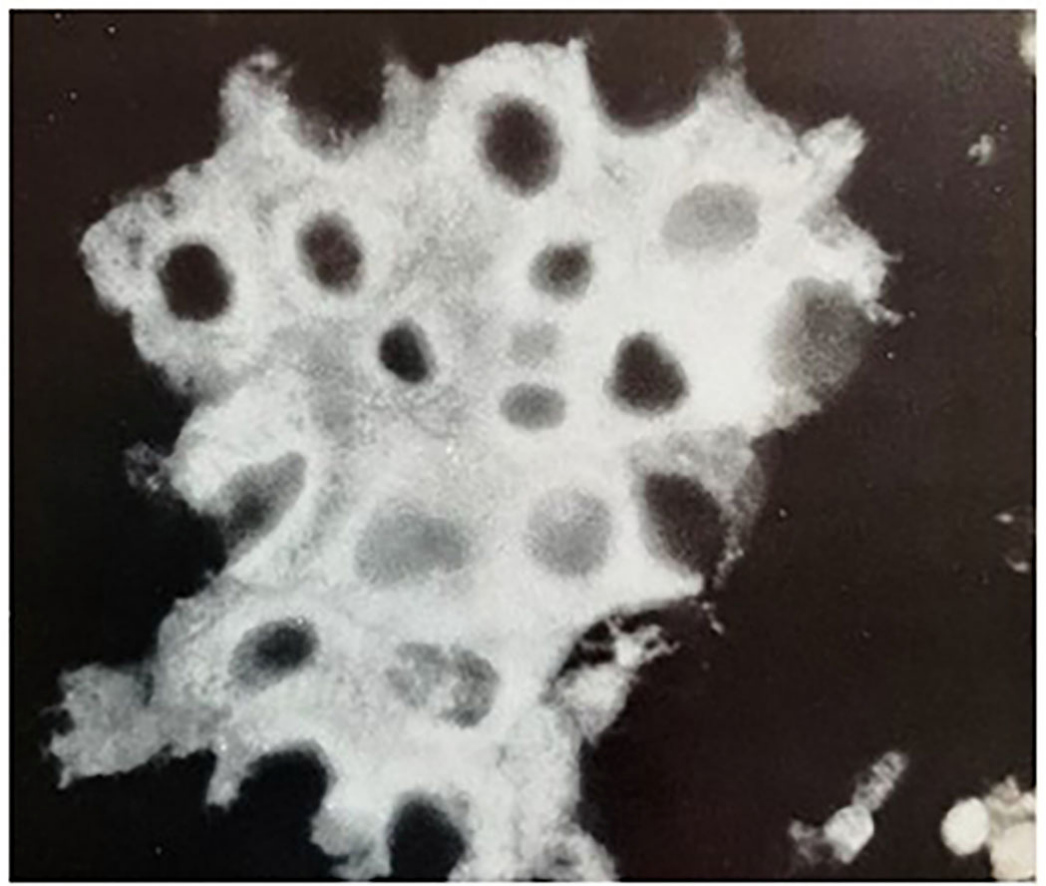
Immunofluorescent localization of AUT-PK 500 in adrenocortical carcinoma 494 cells. Indirect immunofluorescence reaction of rabbit anti-AUT-PK 500 IgG with fixed cells that were freshly isolated from rat adrenocortical carcinoma demonstrated the perinuclear concentration of the enzyme as well as its homogenous cytoplasmic distribution. (Reproduced with permission, Ganguly et al., 1984).

Its immunofluorescent localization in the adrenocortical carcinoma 494 cells is shown in the figure below (Figure 4).

Thus, the elevated expression of the AUT-PK 500 is reminiscent of neoplasia. It became a marker for its detection (United States Patent No. 4,594,319, Issued June 10, 1986, Protein Kinase Enzyme AUT-PK 500 and a Radioimmunoassay for Detection of Neoplasia).

## Two

Ectopic development of adrenergic receptors. Prior to the studies by our group that the adrenocortical carcinoma 494 embodies a novel ectopically-developed α_2_-adrenergic receptor locked in with the membrane guanylate cyclase signal transduction system, it had been demonstrated that this tumor is linked with the ectopically developed epinephrine-dependent β-adrenergic receptor by the Lefkowitz's group (Williams et al., 1977; reviewed in Sharma, 1985). We proved that the α_2_-adrenergic receptor-locked-in with the membrane guanylate cyclase signal transduction system is also modulated by epinephrine. Thus, by two independent signaling pathways, epinephrine modulates these autonomous pathways in the cancer cells. None of these pathways is present in the normal cells.

Below is recounted the establishment of the α_2_-adrenergic receptor-locked membrane guanylate cyclase transduction system at the Pharmacological, Biochemical, and Molecular levels. Thus, the tumor contains two autonomously regulated signaling systems, both epinephrine-modulated, one mediated by the adenylate cyclase and the other by the membrane guanylate cyclase. Historically, it was the first demonstration that such a membrane guanylate signaling system exists in any mammalian cell. And, it still remains unknown in the normal cells. The task involved multiple steps. In all experiments, the isolated adrenocortical carcinoma 494 cells were the subject of investigation.

Step 1, Enzymatic and pharmacological characterization (Perchellet and Sharma, 1980; Shanker and Sharma, 1980). The epinephrine-modulated increase in cyclic GMP is blocked by the α-adrenergic antagonist phentolamine, yet not by the β-adrenergic antagonist propranolol. The rise of cyclic GMP level achieved is additive with that occurring with epinephrine. Thus, the cancer cell inherits two distinct cyclic GMP signaling pathways, one ACTH and the other epinephrine-dependent; yet, only the latter is coupled with the α-adrenergic receptor.

To jump to this critical decision, it needed the firm direct backup studies, as defined below (Perchellet and Sharma, 1980; Shanker and Sharma, 1980).

Mimicking epinephrine, the α-adrenergic agonist, phenylephrine, caused the concentration-dependent rise of cyclic GMP in the adrenocortical carcinoma 494 cells; the rise was blocked by its antagonist, phentolamine. Neither, acetylcholine, a cholinergic agonist, nor calcium caused any rise in cyclic GMP. Importantly, the rise maximally activated by ACTH was additive with that caused by the epinephrine. These results clearly dissected and showed that the adrenal cancer cell possesses ectopic α-adrenergic receptors and that epinephrine causes a rise in the cyclic GMP level through these receptors. They also affirmed the conclusion that the receptors for ACTH and epinephrine are distinct.

This was the first complete enzymatic and pharmacological analysis of the carcinoma cells and their bondage to the guanylate cyclase system.

Then, the studies with the non- and solubilized receptors moved on to settle that they were exclusive of the α_2_-adrenergic receptor subtype; to that date, this subtype was not known. The experiments employed the use of a specific α_2_-adrenergic agonist para-[^3^H]aminoclonidine (PAC), and the α_2_-adrenergic antagonist, yohimbine (Nambi et al., 1982a,b, 1983).

It was, thereby, established that the epinephrine-activated rise in cyclic GMP is mediated by the α_2_-adrenergic receptors, it is coupled with the guanylate cyclase, and the transmembrane signal induced by the α_2_-adrenergic agonist occurs by its interaction with the α_2_-adrenergic receptor, this, in turn, results in the rise of cyclic GMP. The conclusion was that it is one of the means of transmitting the catecholamine-sensitive biological response in the adrenocortical carcinoma 494 cells.

To elucidate the mechanism at the pharmacological, biochemical, and molecular levels by which adrenocortical carcinoma α_2_-adrenergic receptor controls the membrane guanylate cyclase activity, it was critical to purify it and clone its mRNA.

## Purification

It was achieved through an affinity chromatography step using a novel para-aminoclonidine-sepharose resin followed by a gel-permeation high-performance liquid chromatographic step. The radio-iodinated-receptor protein was homogeneous as evidenced by SDS-PAGE gel electrophoresis and by high-performance liquid chromatography. The protein was monomeric in nature with a M_r_ of 64,000 (Jaiswal and Sharma, 1985).

## Pharmacology

It manifested the typical binding characteristics of α_2_-adrenergic receptor, displacement of [^3^H] para-amino-clonidine binding from the receptor by various adrenergic agonists and antagonists. Almost identical biochemical characteristics were then demonstrated in the human platelets (Jaiswal et al., 1989). Both the rat carcinoma and the human platelet forms were monomeric, 64,000-dalton proteins, with a pI of 4.2 and both showed typical α_2_-adrenergic receptor binding characteristics. Nonetheless, the two-dimensional peptide maps of the trypsin-treated proteins showed minor, yet distinct, differences. It was possible that these were the species-specific, rodent vs. human differences. To assess this, the α_2_-adrenergic receptor was cloned.

## Molecular Cloning and Expression

It was achieved using the human platelet α_2_-adrenergic receptor cDNA and rat genomic library. The major difference between the rat and the human forms resided in a stretch of 48-aminoacids within the third cytoplasmic loop. There, the identity was only 45%, demonstrating their differences in terms of their primary amino acid and the predicted secondary and tertiary structural features. The rat brain cDNA was expressed in a mammalian COS-2A cell line. It bound the α_2_-adrenergic antagonist [^3^H]-yohimbine. Based on the binding affinity for prazosin, the cloned receptor was closer pharmacologically and in molecular structure to its human form, α_2A_-adrenergic receptor. Later studies demonstrated that these two forms were species-specific homologs, the one existing in the rodent rat was α_2D_-adrenergic receptor subtype and the human form was α_2A_ subtype, settling that the α_2D_-adrenergic receptor cA2-47 gene was nonexistent in the human genome. Furthermore, besides existing in the rat adrenocortical carcinoma, it existed in the rat tissues of the adrenal gland, brain, testes, and bovine retina. In the latter case crossing the evolutionary ladder (Chalberg et al., 1990).

There were several novel revelations brought forth with this study. One, this was the first cloned rodent α_2_-adrenergic receptor. Two, it represented a new subtype, α_2D_. Three, despite its very close structural identity with its human form, both being the seven-trans-membrane-spanning proteins, biochemically, it was uniquely linked with the membrane guanylate cyclase signaling system.

## Conclusions in Relevance to Cancer

This presentation demonstrates two important characteristics of a malignant cell. One, it grows without regulation by the cyclic GMP control signals present in the normal cells. Two, it possesses the property of metastasis. In addition, a typical characteristic of many adrenocortical carcinomas is their loss of regulation by ACTH in the synthesis of steroid hormones. Gaining an understanding of this loss requires precise knowledge of the ACTH-modulated machinery in its normal counterpart. The studies reviewed here have not addressed the question of whether the achievement of hormonal independence of adrenocortical cells is in any way related to malignancy. However, it can be speculated that the initial pre-neoplastic lesion in an adrenocortical cell results in the expression of a gene that is normally suppressed. This gene product, in turn, causes the transformation of the normal to the neoplastic cell. This speculation is in accordance with the multistep concept of neoplasia, in which the first event is immortalization, which subsequently leads to the neoplastic cell. The speculation is in accordance with the current notion that normal cells contain proto-oncogenes which under certain conditions can be activated to oncogenes, resulting in the initiation of/or sustaining carcinogenesis. It is then possible that (i) AUT-PK 500 is an adrenocortical cellular oncogene; (ii) the protein kinase is a cause for either immortalization or its subsequent steps in the adrenal cell; (iii) low-level expression of the enzyme is necessary for cellular growth with differentiation, but it is only when the gene expression is excessive that the cell acquires cancer phenotype (Reviewed in Sharma et al., 1988).

Broadening this concept to other cancers, it is in accordance with the original proposal that protein kinases complexed with the mRNAs, mRNP particles, play a key role in the translational processes of the liver and the hepatomas. The original observation demonstrated that the protein kinase of membranes of the endoplasmic reticulum of highly differentiated hepatomas has a different orientation within the membrane from that of the same enzyme found in the endoplasmic reticulum of the liver (Sharma et al., 1976). This difference may thus make it unavailable for phosphorylation of certain proteins in the tumor that are normally phosphorylated by this enzyme in the liver.

## Author Contributions

The author confirms being the sole contributor of this work and has approved it for publication.

## Conflict of Interest

The author declares that the research was conducted in the absence of any commercial or financial relationships that could be construed as a potential conflict of interest.

## Publisher's Note

All claims expressed in this article are solely those of the authors and do not necessarily represent those of their affiliated organizations, or those of the publisher, the editors and the reviewers. Any product that may be evaluated in this article, or claim that may be made by its manufacturer, is not guaranteed or endorsed by the publisher.
